# A hybrid machine learning framework to improve prediction of all-cause rehospitalization among elderly patients in Hong Kong

**DOI:** 10.1186/s12874-022-01824-1

**Published:** 2023-01-13

**Authors:** Jingjing Guan, Eman Leung, Kin-on Kwok, Frank Youhua Chen

**Affiliations:** 1 Epitelligence, Hong Kong SAR, China; 2 JC School of Public Health and Primary Care, The Chinese University of Hong Kong, Hong Kong SAR, China; 3 Stanley Ho Centre for Emerging Infectious Diseases, The Chinese University of Hong Kong, Hong Kong SAR, China; 4 Hong Kong Institute of Asia-Pacific Studies, The Chinese University of Hong Kong, Hong Kong SAR, China; 5 Department of Management Sciences, City University of Hong Kong, Hong Kong SAR, China

**Keywords:** Rehospitalisation prediction, Temporal risk estimator, Hybrid machine learning, Electronic health records, Predictive Modelling

## Abstract

**Background:**

Accurately estimating elderly patients’ rehospitalisation risk benefits clinical decisions and service planning. However, research in rehospitalisation and repeated hospitalisation yielded only models with modest performance, and the model performance deteriorates rapidly as the prediction timeframe expands beyond 28 days and for older participants.

**Methods:**

A temporal zero-inflated Poisson (tZIP) regression model was developed and validated retrospectively and prospectively. The data of the electronic health records (EHRs) contain cohorts (aged 60+) in a major public hospital in Hong Kong. Two temporal offset functions accounted for the associations between exposure time and parameters corresponding to the zero-inflated logistic component and the Poisson distribution’s expected count. tZIP was externally validated with a retrospective cohort’s rehospitalisation events up to 12 months after the discharge date. Subsequently, tZIP was validated prospectively after piloting its implementation at the study hospital. Patients discharged within the pilot period were tagged, and the proposed model’s prediction of their rehospitalisation was verified monthly. Using a hybrid machine learning (ML) approach, the tZIP-based risk estimator’s marginal effect on 28-day rehospitalisation was further validated, competing with other factors representing different post-acute and clinical statuses.

**Results:**

The tZIP prediction of rehospitalisation from 28 days to 365 days was achieved at above 80% discrimination accuracy retrospectively and prospectively in two out-of-sample cohorts. With a large margin, it outperformed the Cox proportional and linear models built with the same predictors. The hybrid ML revealed that the risk estimator’s contribution to 28-day rehospitalisation outweighed other features relevant to service utilisation and clinical status.

**Conclusions:**

A novel rehospitalisation risk model was introduced, and its risk estimators, whose importance outweighed all other factors of diverse post-acute care and clinical conditions, were derived. The proposed approach relies on four easily accessible variables easily extracted from EHR. Thus, clinicians could visualise patients’ rehospitalisation risk from 28 days to 365 days after discharge and screen high-risk older patients for follow-up care at the proper time.

**Supplementary Information:**

The online version contains supplementary material available at 10.1186/s12874-022-01824-1.

## Introduction

Hospitalisation amongst older adults is common [1], prolonged [2], avoidable [3] and often results in rehospitalisation [4] compared with that of their younger counterparts. As the global population ages rapidly [5], the disease burden that an ageing population imposes on the healthcare system also exacerbates [6]. Predicting older patients’ rehospitalisation risk could benefit clinical decisions and service planning. However, studies predicting probability of rehospitalisation focus primarily on the post-discharge timeframe of 28 or 30 days, and predictors such as patients’ diagnostic and clinical profiles and the care quality received prior to discharge [7]. Moreover, the performance of the published models of 28-day (or sometimes 30-day) rehospitalisation is generally modest, with only a few notable exceptions [7]. No statistical difference has been observed between the performance of regression-based models and applied machine learning (ML, mean c-statistics of 0.74 vs. 0.71) [7] even though ML generally outperforms traditional statistical models [8–14].

The modest performance of models that use patients’ acute diagnoses and clinic profiles as predictors deteriorates quickly when the timeframe of rehospitalisation prediction goes beyond 28 days post-discharge. Similarly, poorer model performance is found in studies with older adults as participants than with only younger ones [15]. In particular, the prediction performance is even poorer when modelling older adults’ rehospitalisation over an extended timeframe (e.g. 1 year) by using only the clinical characteristics of patients in an acute care setting as the predictors [16, 17]. Meanwhile, some studies considered older adults’ rehospitalisation over an extended timeframe by using predictors related to functionality and dependency [18–22]. Frequent hospitalisations were often defined as having two or more episodes of hospitalisations within a year [21–25]. The predictors based on which rehospitalisation over an extended timeframe is modelled are momentous measures of one’s level of deterioration rather than patients’ clinical and diagnostic statuses; the performance of these models was also modest, with an average c-statistics of 0.69 [26–39].

In sum, the literature showed that the performance of modelling older adults’ rehospitalisation is modest for a 28- to 30- day prediction after discharge, and the model performance deteriorates for a longer-range prediction, after controlling the effect of diagnostic/clinical/functional profiles of patients and care quality patients received prior to discharge. To the authors’ knowledge, neither the frequently-used regression-based models nor the ML models in the literature have accounted for any temporal dimension [7, 40, 41]. To this end, the present study aimed to develop a model for estimating elderly rehospitalisation risk within any timeframe under 1 year by accounting for older adults’ deterioration over time. With the model developed here, risk estimators were derived, and the proposed approach was validated retrospectively and prospectively. The proposed model’s effect was also iteratively compared against the effects of any post-acute, ambulatory and residential care received after discharge by hybridising the risk estimator with an ML model [42, 43]. Notably, it has been shown that post-acute and ambulatory services received post-discharge are less likely to be rehospitalised, and older adults are more likely to have received convalescent and ambulatory care upon discharge [44, 45]. By contrast, older residential care recipients are more likely to be hospitalised than community -dwelling order adults [46]. However, to the authors’ knowledge, none of the published models on rehospitalisation prediction accounted for the effects of post-acute, ambulatory and residential care that elderly patients received post-discharge.

## Method

This study is based on data extracted from electronic health records (EHRs) of older patients (60+) admitted to the medical ward (internal medicine) of the study hospital (a 1900-bed acute tertiary hospital in Hong Kong) between 2014 and 2017. For the purpose of model building, we tracked the rehospitalisation episodes of a cohort of community-dwelling patients who were discharged in 2014 for at least 1 year, with the last discharge of the cohort tracked until Dec 31, 2015. It is of note that community-dwelling patients, unlike residents of residential care homes, had no around-the-clock professional care provided post-discharge. After having developed the model, the model was validated retrospectively and prospectively. In retrospective validation, the model was tested in an out-of-sample cohort whose outcomes of interest were already known at the time of the study. By contrast, prospective validation examined the model’s accuracy by predicting an outcome that was unknown at the time of prediction and only verified the prediction when the outcome had become known. Here, for the purpose of prospective validation, data were collected after the proposed model was piloted as part of the clinical operations of the study hospital. More specifically, retrospective validation was carried out on an out-of-model sample of community-dwelling patients discharged in 2015 (from January to December). Their rehospitalisation episodes were tracked for a year, with the last discharge of the retrospective validation cohort tracked until Dec 31, 2016. Prospective validation was conducted monthly on 12-month cohorts discharged in 2016 from the study hospital.

Cohorts for model validation versus model training were split to avoid overlaps in admission records, individual patients, or timeframes. For example, models for estimating risk should reflect the “intended clinical use” [74]. The proposed model’s intended clinical use is to enable decision-makers to estimate the future rehospitalisation risk of the current cohort of patients about to be discharged. Thus, this study has taken the more challenging task of testing the model with data sampled from a subsequent period rather than contemptuous ones.

No statistical difference was found between the model-building and model-validation datasets concerning the following metrics: the 28-day rehospitalisation rates were 21.6 and 20.5% in the model-building and model-validation datasets, respectively; the corresponding averages of acute length of stay (LOS) were 6.3 and 6.0, respectively; the intensive-care-unit (ICU) admission rates were 0.304 and 0.342%, respectively; and amongst those admitted to the ICU, the average ICU LOSs were 5.6 and 8.3 days, respectively. The prevalence of having ever been diagnosed with chronic illnesses in the model-building and model-validation datasets were as follows: cancer: 7.1% versus 7.3%; COPD: 21.5% versus 20.0%; stroke: 26.4% versus 23.7%; and diabetes: 26.2% versus 23.7%. The probability distribution of the validation dataset was as follows: the 28-, 30-, 60-, 90-, 120-, 150-, 180-, 210-, 240-, 270-, 300-, 330- and 365-day rehospitalisation rates were 20.5, 21.5, 30.7, 36.3, 40.1, 43.0, 45.6, 47.8, 49.8, 51.4, 52.9, 54.2 and 55.6%, respectively.

In addition to validating the proposed model retrospectively and prospectively, we iteratively compared our estimator for rehospitalisation risk against the effect of post-acute, ambulatory, and residential care on 28-day rehospitalization in a sample of patients admitted to the study hospital in 2017. Unlike the model-building and model validation cohorts, the cohort for testing the risk estimator against the effects of other post-discharge services different patients received included community-dwelling elderly and residential care home residents.

The rest of the method section describes the development of the model from which the risk estimator was derived and the hybrid ML model through which the conditional inference of the risk estimator was made against the effect of other post-discharge services.

### Building a temporal zero-inflated Poisson (tZIP) model

In the current study a zero-inflated Poisson (ZIP) model with four predictors and a temporal offset function was built to estimate the likelihood of rehospitalisation within any timeframe under 1 year.

Firstly, ZIP model was chosen here to ensure the robustness of our analysis despite the excessive zero counts in our rehospitalisation data. Secondly, in terms of predictors, length of stay, acuity of admission, Charlson comorbidity score [47, 48] and the number of Emergency Room (ER) visits in the previous 6 months were chosen because these four factors (collectedly known as the ‘LACE’ [49]) are often regarded as the ‘gold standard’ in informing interventions to reduce rehospitalisation across clinical settings [9]. The LACE is one of the most validated sets of risk factors for rehospitalisation [40, 50]. Below, the LACE score was not standardised as LACE is already a validated instrument and the unstandardised score afforded the LACE-based modelling result to be more interpretable. Finally, a temporal offset functions were included in the ZIP model (hence, tZIP) to account for the deterioration of the aging population over time. Below, the parameter derived from the offset function was standardised into a value between 0 and 1 to parameterise the proportion of the 2-year study period each record’s offset term represents. Since the standardisation here represented only a linear transformation of the natural time scale, only in-sample data were used to standardise the offset terms during model building and validation.

Please refer to the supplementary material for tZIP model’s mathematical formulation and the derivation of the model’s joint estimator (JE).

We used R to analyse the data. The tZIP model was examined using the R package ‘pscl.’ The R package ‘pROC’ was used to generate the area under the receiver operating curve (AUC) and the area under the precision-recall curve (AUPRC) for measuring performance, and ‘ggplot2’ was used to generate plots. AUPRC is particularly sensitive to positive cases (i.e. readmission) when the data are highly imbalanced.

### Prospective validation

In addition to validating our model with a retrospective cohort, we have also performed a prospective validation. Specifically, after our model was implemented as part of the actual clinical operation, we prospectively compared our model’s prediction against the rehospitalisation outcome of monthly discharged cohorts tracked in real-life. While rarely performed, prospective validation can enable better integration of research into clinical practices and a more accurate evaluation of the research’s direct impact on patient care [51]. Here, prospective validation was performed monthly on 12 monthly cohorts of community-dwelling patients discharged in 2016 from the study hospital. The EHR of the study hospital was subsequently reviewed until December 2017 to assess if the patients were rehospitalised within 28 days following their discharges.

### Hybrid ML method for validating the JE in tZIP against the effects of post-acute, ambulatory and residential services patients received after discharge

In addition to prospective validation, a hybrid ML method was introduced to compare the contribution of our risk estimator to the 28-day rehospitalisation with the individual and collective effects of patient clinical profiles and his/her utilisation of health services on the 28-day rehospitalisation. Here, patient clinical profiles consist of features representing one’s diagnoses, comorbidity, intervention procedures, and ICU or surgical events [38]. On the other hand, the services whose utilization was of interest include acute, post-acute, ambulatory, and residential care offered by the medical system studied here. The comparisons were conducted iteratively via conditional inferences. The objective of applying the hybrid ML method was to test the hypothesis that the JE’s contribution to 28-day rehospitalisation outcomes was greater than, and independent from, the unique or combined contributions of all other clinical and utilisation-related features. Hence, the result of hybrid ML was reported separately from the retrospective and prospective validations. As the objective of our retrospective and prospective validations was to examine the performance of JE in predicting monthly cohort’s rehospitalization outcomes among community-dwelling elderly alone.

In literature, hybridisation between a linear model and an ML model is performed to improve ML models’ generalisability [42, 43], performance [42, 52–54] and interpretability [55]. Here, the purpose of hybridisation is instead to leverage the ML model’s unique ability to compare the marginal contribution of each feature to all others in the pool and test the hypothesis that the predictability of the risk estimator is greater than, and independent from, the effects of post-acute, ambulatory and residential care patients received post-discharge. Rather than hybridising with a linear model, the ML model in the current study was hybridised with a ZIP regression estimator [56] with a mixture of probability functions [57] due to the excessive zeros and a long tail resulting from the low prevalence of rehospitalisation events over time.

In addition, Unbiased Recursive Partitioning with Surrogate Splitting (URPSS) [58] method was applied in our hybrid ML model to compare the marginal contributions of the estimators and all the different services patients received post-discharge or sometimes received concurrently. The following characteristics of URPSS aligned with the study’s objective and provided URPSS with an edge over other partitioning methods of the decision tree [59]. First, splitting along the decision tree does not take place in isolation for URPSS; instead, each feature is recursively compared with every other feature in the pool to make conditional inferences of the effect of each feature on the outcome. Second, URPSS’ global optimisation allows features to be selected in an unbiased manner and consequently, the overfitting of data is minimised in partitioning. Third, in addition to data missing randomly, URPSS could handle logically (and thus systematically) missing data, which are abundant amongst services offered post-discharge. For example, if a patient is not eligible to receive a service, data on specific aspects of receiving services, such as the timing and duration, are coded as missing. Please refer to the supplementary material for a detailed description of, and a schematic on, the URPSS process.

## Result

### Model building

A total of 18,805 index hospitalisations in the model building between Jan 1, 2014, and Dec 31, 2014, were included. Table 1 presents the tZIP-relevant statistics. The average LOS per index hospitalisation was 6.4 days (SD = 7.4). The Charlson comorbidity index was on an average of 1.0 (SD = 1.4), and the average number of ER visits within 6 months was 0.8 times (SD = 1.2), with a median of zero ER visits within 6 months. Specifically, the model building sample’s average exposure time (time between discharge and subsequent rehospitalisation) was 448.7 days (SD = 186.5), with an average rehospitalisation count of 1.6 times (SD = 2.3) and the first quartile being zero.

**Table 1:** Descriptive statistics of predictors (LACE), exposure time, and rehospitalisation outcomes in the training dataset

Variable	Mean (SD)	Min	First Quartile	Median	Third Quartile	Max
Exposure Time, d	448.7 (186.5)	0.3	340.4	340.4	601.4	728.2
Rehospitalisation, No.	1.6 (2.3)	0	0	1.0	2.0	41.0
**L**ength of Stay, d	6.4 (7.4)	0.1	2.9	4.6	7.3	315.2
**C**harlson Comorbidity Index	1.0 (1.4)	0	0	0	1.0	13.0
Visits to **E**mergency Room during Previous 6 Months, No.	0.8 (1.2)	0	0	0	1.0	13.0
Prevalence of **A**cute Admission	87.7%					

Notwithstanding the disproportional amount of zeros in the response variable, the ZIP model estimated that 59.3% of the zero rehospitalisation actually belonged to the Poisson distribution (*λ*), representing at-risk patients whose zero rehospitalisation could turn positive if given time (i.e. “active”). Meanwhile, the remaining 40.7% of zero rehospitalisation were considered “inactive”, i.e., belonging to the binomial distribution (*p*). When selecting a temporal offset function for the tZIP model, the function where *p* is convexly decreasing and *λ* is concavely increasing as post-discharge time increases yielded a model that fitted the training dataset best and was used in the analyses reported in the following.

Table 2 demonstrates the estimated coefficients and the corresponding odds ratios (ORs) and rate ratios (RRs) from the logistic (with a distribution of *p*) and Poisson (with a distribution of *λ*) components of the tZIP model. For the logistic component, the presence/high score of the four factors of LACE is negatively associated with one’s rehospitalisation being ‘inactive’ (i.e., with a rehospitalisation probability of *p*). Hence, greater likelihood of rehospitalisization entailed. Especially, the number of ER visits during the past 6 months (OR = 0.627, *P*-value <2E-16) and the index hospitalisation being acute (OR = 0.627, *P*-value <2E-16) have the most significant effect that triggers rehospitalisation, followed by a high score on the Charlson comorbidity index (OR = 0.971, *P*-value = 8.59E-03) and an extended length of stay during the index hospitalisation (OR = 0.988, *P*-value = 1.93E-05). Meanwhile, a mix of effects of the four factors of LACE could be observed on the expected rehospitalisation if an index hospitalisation is in active rehospitalisation status (i.e., with a rehospitalisation probability of *λ*) As shown in Table 2, the number of ER visits in the past 6 months (RR = 1.161, *P*-value <2E-16) and the Charlson comorbidity index (RR = 1.054, *P*-value <2E-16) were positively associated with the expected number of active rehospitalisation. By contrast, the longer length of stay during the index hospitalisation was associated with less expected rehospitalisation (RR = 0.995, *P*-value = 3.82E-09), whilst the more acute the index hospitalisation was, the less rehospitalisation could be expected (RR = 0.812, *P*-value <2E-16).

**Table 2:** Odds ratios and rate ratios of the logistic and poisson components of the tZIP Model

**Probability of Inactive Rehospitalisation Status (*p*(*t*))**	**Odds Ratio**	**Coefficient (SE)**	**z Value**	***P*** **Value**	**Sig.**
Intercept	2.385	0.869 (0.043)	20.058	< 2E-16	***
Length of Stay	0.988	−0.012 (0.003)	−4.272	1.93E-05	***
Acute Admission (Yes)	0.627	−0.467 (0.041)	− 11.306	< 2E-16	***
Charlson Comorbidity Index	0.971	−0.029 (0.011)	−2.628	8.59E-03	**
Visits to Emergency Room during Previous 6 Months	0.627	−0.467 (0.018)	−25.955	< 2E-16	***
**Probability of Active Rehospitalisation Rate (*λ*(*t*))**	**Rate Ratio**	**Coefficient (SE)**	**z Value**	***P*** **Value**	**Sig.**
Intercept	1.061	0.059 (0.016)	3.679	2.34E-04	***
Length of Stay	0.995	−0.005 (0.001)	−5.892	3.82E-09	***
Acute Admission (Yes)	0.812	−0.208 (0.015)	− 13.706	< 2E-16	***
Charlson Comorbidity Index	1.054	0.053 (0.003)	15.216	< 2E-16	***
Visits to Emergency Room during Previous 6 Months	1.161	0.149 (0.003)	48.291	< 2E-16	***

### Model validation

The tZIP model was validated using the 2015 cohort’s hospitalisation records (*n* = 15,055) that were not included in the model building. Considering the care and resource planning was conducted periodically within 1 year, the annual cohort was divided into 12 subsets on the basis of each record’s month of admission, with the data extraction day (i.e., end-of-observation date) being the *r*^th^ day after the average discharge time of the subset’s observations. Subsequently, the proposed approach’s accuracy in predicting the 30-day and longer-term (up to 365-day) rehospitalisation was evaluated. In parallel, the performance (parameterised as AUCs and AUPRCs) of Cox’s proportional hazard model (Cox model hereafter) and the traditional LACE score model (Linear model hereafter) were compared; both shared the same predictors as the tZIP model.

**Fig. 1:**
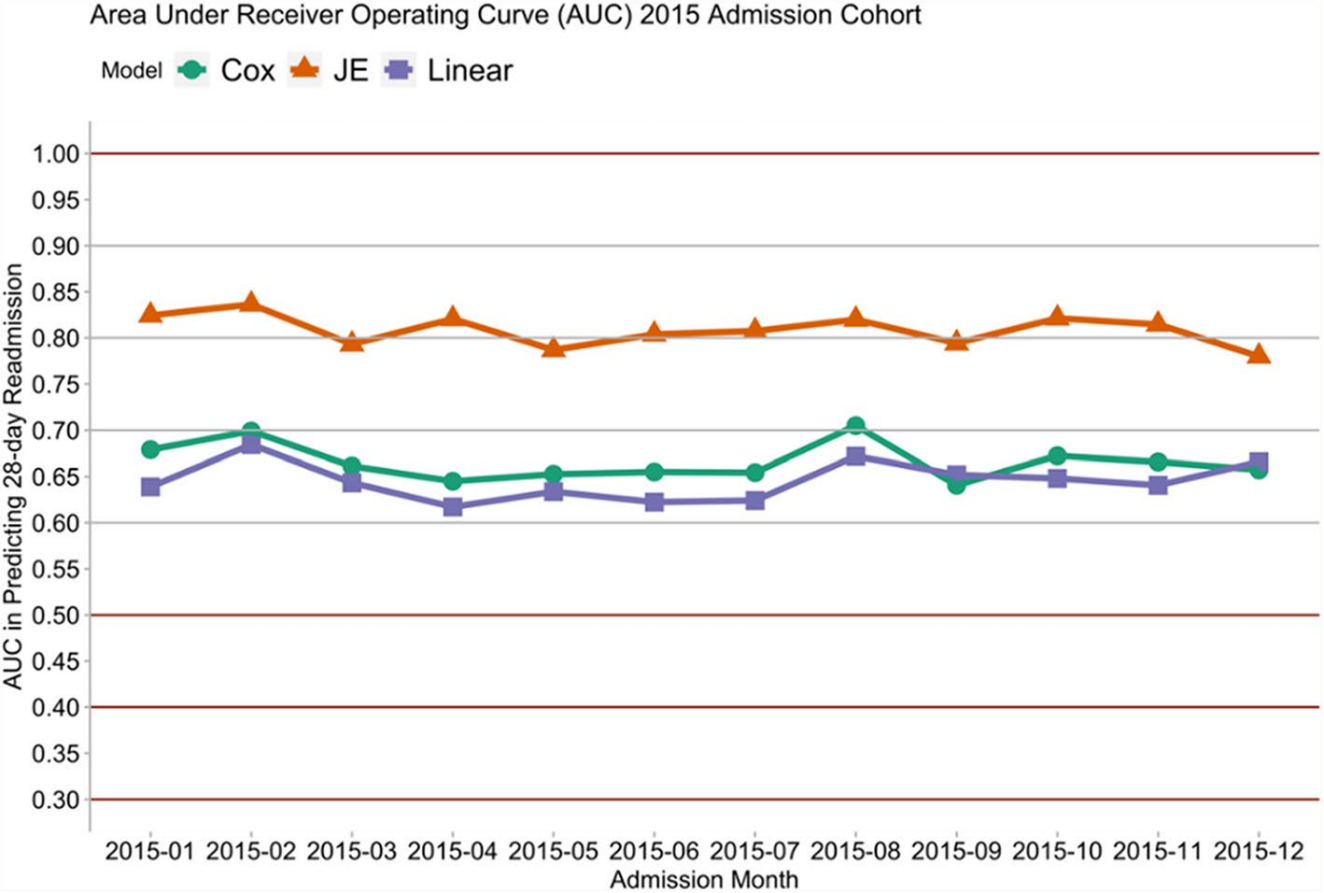
Comparing AUCs of 28-day rehospitalisation predictions: linear model, Cox’s proportional hazard model and JE

**Fig. 2:**
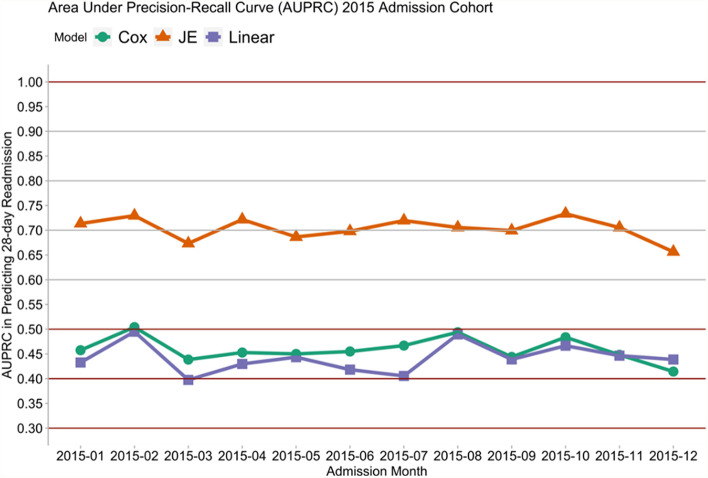
Comparing AUPRCs of 28-day rehospitalisation predictions: linear model, Cox’s proportional hazard model and JE

**Fig. 3:**
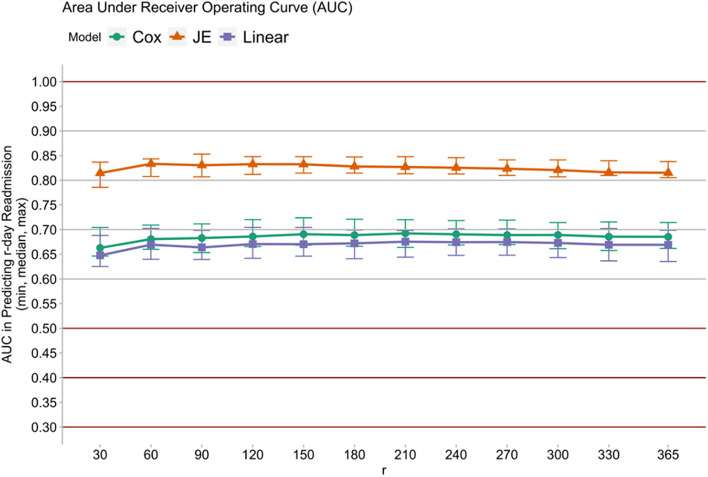
Comparing AUCs of longer-term rehospitalisation predictions: linear model, Cox’s proportional hazard model and JE

**Fig. 4:**
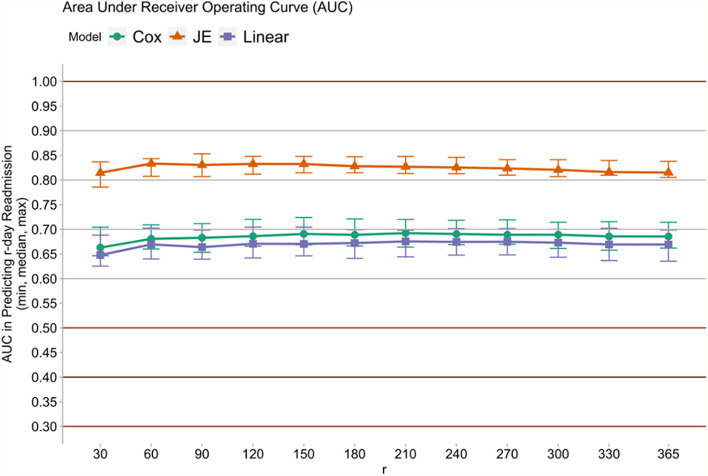
Comparing AUPRCs of longer-term rehospitalisation predictions: linear model, Cox’s proportional hazard model and JE

Figures 1, 2, 3 and 4 show that the JE outperformed the Cox and Linear models. In particular, the orange line in Figs. 1 and 2 showed that the JE outperformed Cox and Linear models in predicting 28-day rehospitalisation, with JE’s AUCs being generally above 80% and AUPRCs around 75%. By contrast, the AUCs of the Cox and Linear models fell between 60 and 70%, and the AUPRCs generally fell around 50% or below. Similarly, Figs. 3 and 4 show that JE outperformed Cox and Linear models in predicting 30-day and longer-term (up to 365-day) rehospitalisation. Whilst JE’s AUCs stayed above 80% between 30 and 365 days, Cox and Linear models’ AUCs hovered around 65% (Fig. 3). Meanwhile, the 30-, 90- and 180-day AUPRCs were 73, 85% and around 90%, respectively, for JE and below 50, 70 and 75%, respectively, for Cox and Linear models (Fig. 4).

**Fig. 5:**
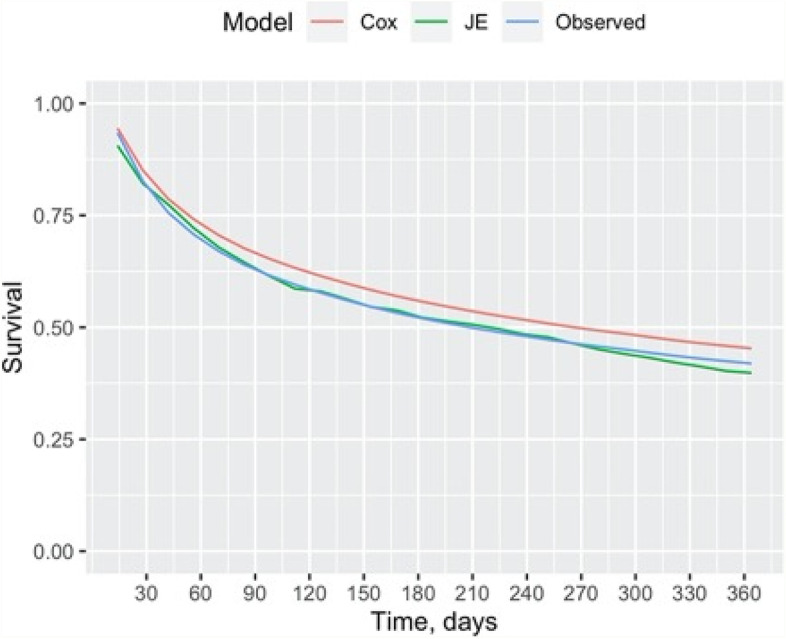
Comparing to survival rates estimated by Cox’s proportionals: hazards model and JE compared with the observed survival rate

In addition to comparing Cox’s model and JE’s performance in predicting the studied cohort’s rehospitalisation outcomes across different timeframes, Fig. 5 reports the cohort’s survival rates by the two models, i.e., the proportion of studied cohort not rehospitalised overtime against the observed survival rate in a Kaplan–Meier plot. As shown in, Fig. 5, the survival rate estimated by JE was much closer to the observed survival rate than Cox’s model’s estimation.

**Fig. 6:**
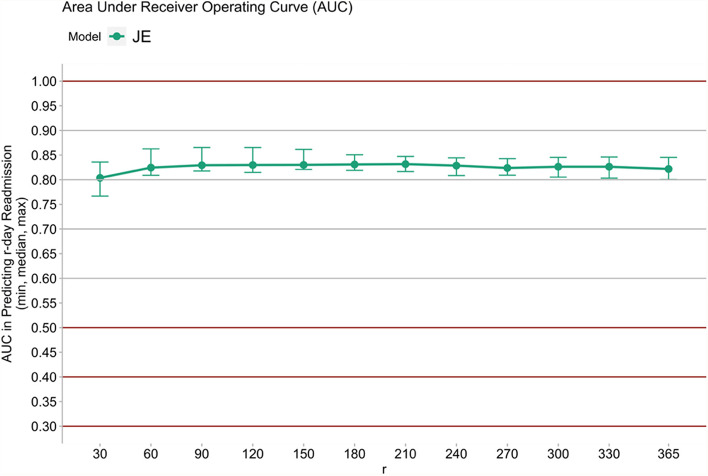
Prospective validation AUCs of JE over a one-year period

Prospective validation was also performed in addition to retrospective validation. As shown in Fig. 6, the performance of the JE in prospective validation consistently stayed above 80%.

### Result from the hybrid ML

The hybrid ML algorithm selected our JE *first* among the entire pool of clinical and service utilisation-related features in a 28-day rehospitalisation outcome-supervised URPSS process (AUC = .78). In other words, JE was selected by the algorithm as the ‘mother node:’ The feature from which all other features were split and on which all other features’ effects were conditionally based. Hence, the finding supported the hypothesis that the contribution of JE on 28-day rehospitalisation was superior to, and independent from, the contribution of all other features, singly or in combination. Consequently, as the objective of applying a hybrid ML algorithm was to test the hypothesis that the JE’s contribution outranks all other features, individual paths that spilt from JE the ‘mother node’ were not shown as they were not the focus of this study.

## Discussion

The rich EHR data provide opportunities to develop temporal risk prediction modelling for large-scale populations. In this study, a mixture model with two temporal components and a model-based joint risk estimator for depicting all-cause rehospitalisation risk over time within any timeframe between 28- and 365-day post-discharge were developed and validated. With AUCs of above 80% in the external validation samples, the proposed approach outperformed most relevant published models with an average AUC of 69% [26–39] and it was on par with the relatively advanced ML models with a median AUC of 68% [60]. Using the same dataset of the tZIP model, the AUCs of Cox and Linear models closely aligned with the literature’s best AUC, between 60 and 70%. The modest AUCs of Cox and Linear models provided evidence that ignoring temporal changes or assuming linear changes in rehospitalisation risk over time led to a poorer fit of older patients’ rehospitalisation patterns. In turn, the good performance, for the first time, confirmed the nonlinearity association between rehospitalisation risk and exposure time for older patients. Such nonlinearity between risk and time could be directly visualised in the exploration of the studied data shown in Additional file 1: Fig. S-1.

This study contributes to an enhanced understanding of the association between time and rehospitalisation risk for older patients. The proposed approach allowed the EHR-based data to choose the type of nonlinearity empirically. Methodologically, generalised linear models could also be time-varying as an alternative to other time-varying models, such as survival models, that were used in literature [61]. Another alternative to handling nonlinear risk changes over time is to solely transform Poisson or logistic regression models to make time-varying predictions at constant rates similar to the Cox model [58, 62]. In the present work, tZIP was found to fit the data better than ZIP, whilst ZIP fitted the data better than the transformed Poisson or logistic regression. This finding affirmed the importance of handling excessive zeros in observed rehospitalisation counts amongst older patients and the nonlinear risk changes over time.

The clinical meaning of the nonlinear association between time and rehospitalisation risk of older adults is no less critical. Rehospitalisation models should be developed separately for older and young patient populations. The identified temporal complexity is possibly driven by the fact that older adults deteriorate more rapidly and nonlinearly than their younger counterparts. It, in turn, explains why poor performance amongst older adult samples than their younger counterparts was observed in time-invariant rehospitalisation prediction models that dominate the literature [15]. Hence, more research is needed.

This study addressed the gap in the literature; that is, previous studies seldom examined in one paper rehospitalisations that take place in different timeframes (say, 28 and 365 days), which makes it difficult to gauge a risk estimator’s performance over different rehospitalisations timeframes. Besides good discrimination in predicting 28-day rehospitalisation, a time-varying estimator of rehospitalisation risk that is flexible in its application to any rehospitalisation timeframe between 28 and 365 days was put forward to extend the temporality of a risk estimator for rehospitalisation. The risk estimators performed consistently better than the Cox and Linear models over the course of 365 days. However, a slight decrease in AUC was observed as the timeframe for predicting rehospitalisation risk widened to 1 year. The fluctuations in the JE’s predictability of rehospitalisation could be attributed to the highly variable post-discharge services and follow-up care the sample may have received between 28 and 365 days after discharge. However, by iteratively comparing the risk estimator with the post-acute, ambulatory, and residential care the elderly patients received post-discharge, the proposed ML model demonstrated that the JE’s superb performance was not affected by patients’ post-discharge service ecology. To the authors’ knowledge, the current study was the first to use hybrid ML to examine the performance of a rehospitalisation prediction model within one’s post-discharge environment.

In fact, whilst patients’ rehospitalisation risk and the clinical decisions informed by the risk estimates are affected by patients’ post-discharge environment, it has not been incorporated as a component in any of the published models on rehospitalisation risk. Notably, Goldstein et al. [63] concluded that the poor performance of risk prediction models is generally attributable to their failure to estimate risk in accordance with the “intended clinical use at the point of clinical decision.” For example, benefiting clinical decisions at the point of discharge planning is the accurate estimation of patients’ rehospitalisation risk and the extent that it could be mitigated by the service ecology to which patients are being discharged. Consequently, a temporal offset function was built into the proposed model to encapsulate the 2-year trajectory of the rehospitalisation-mitigating effect of the regional population’s service ecology. In addition, the built model was validated by comparing the marginal predictability of JE against the acute, post-acute, ambulatory, and residential care in the patient’s post-discharge service ecology.

Using EHR data brought a similar disadvantage shared by previous studies that certain variables are not collected within the EHR [57]. The cohorts employed in the present study were older Chinese patients discharged alive from the hospital in Hong Kong. The direct modelling results should apply to all such patient populations in Hong Kong. However, the medical services differences by geographical areas remain unstudied in current Hong Kong, which may also affect the applicability of the results. The estimation results may not be generalisable to other Chinese older patient populations outside of Hong Kong or non-Chinese older patients. Researchers could replicate the entire design with their EHR data and contexts regarding these theoretically inapplicable samples. The EHR data for the model-building and the model-validation cohorts were sampled consecutively from the same medical ward, possibly increasing the validation AUCs. Research with EHR data could hardly obtain a completely external sample to validate a model but the use of an out-of-model sample could be a solution to the issue.

The proposed approach assumed that the clinical and functional declines of the selected older adults population remained the same during the study period, which could be unrealistic in a rapid aging context. To offset this assumption’s potential adverse effect, rehospitalisation events over 2 years were used as an omnibus proxy measure to capture the deterioration of the study population. For example, in the literature on frequent hospitalisations, the researcher measured participants’ ability to live independently in the community to assess their level of deterioration [64]. However, studies published thus far relied only on one-time measures of the participants’ ability to independently engage in activities of daily living as an assessment of their decline and deterioration. Zhao et al. [65] stated that rehospitalisation risk models should be based on ‘all discharges as opposed to just the first discharge per patient and utilise methods that account for clustered data.’ Deterioration and decline are, by definition, temporal constructs that include previous care utilisation, chronicity and temporal model formulation to control. Future studies could have more measures on functional status and one’s environment for stable factors that continuously affect one’s rehospitalisation risk. The unspecific effects of post-acute and residential care on rehospitalisation were considered in this study, and they showed to be secondary to the proposed estimator.

Institutionalised older patients accounted for around 40% of elderly utilisation in the study hospital. Their hospital utilisation also affects the allocation of medical resources to the community-dwelling older adults. Previous studies did not target this co-existing population when studying community-dwelling patients, possibly due to limited data availability. To address this limitation, a rehospitalisation estimator was validated in a cohort with community-dwelling elderly and long-term residential care residents. Similar to the hybrid ML validation, the temporal estimator’s contribution to 28-day rehospitalisation prediction remained outweighed the contribution of patients’ discharge location.

However, the model’s overall performance deteriorated from greater than 80% AUC to 78% AUC after including residential care patients. In fact, the model performance deteriorated despite the application of ML and a comprehensive feature pool that includes patients’ clinical profiles and their post-discharge environment captured on EHRs. Such deterioration could be attributed to factors not captured by EHR, such as the quality of care provided in different long-term care facilities to patients discharged and patients’ functional and psychosocial challenges, which could be improved in future research if more critical non-EHR information could be collected.

## Conclusions

With the zero inflation and dual-parameter temporal components in predicting rehospitalisation counts within a 2-year exposure time, a new rehospitalisation risk model and its risk estimators that accounted for the nonlinear post-discharge deterioration were proposed and validated. The approach outperformed the estimations conducted with time-invariant or rate-invariant models, especially in an extended rehospitalisation timeframe. The good discriminations of the time-varying estimation of rehospitalisation risk were not affected by the chronic and complex conditions that characterised elderly hospitalisations. The time-varying risk estimator was the prominent factor amongst the diverse post-acute care a patient may receive due to his/her conditions at discharge. The proposed approach also relied on four LACE variables that could be easily computed from EHR systems and allowed clinicians to visualise a patient’s rehospitalisation risk from 4 weeks to 365 days since discharge. This new approach is useful in screening and identifying high-risk older patients for proper follow-up care at the proper time, which shall benefit healthcare systems in clinical, policy and operational aspects if adopted in practice.

## Supplementary Information

**Additional file 1.**

## Data Availability

The Hong Kong Hospital Authority owns the data, and hence we cannot share it publicly. The datasets generated and/or analysed during the current study are not publicly available due to restrictions being put on data sharing by Hong Kong’s Personal Data (Privacy) Ordinance (Cap. 486) (PDPO), including, but not exclusive to, PDPO’s Guidance Note in Cross-border Data Transfer. In addition, the Research Ethics Committees of the Hospital Authority do not allow a third-party transfer of patient data. Nor do the Ethics Committees permit study PI to make public EHRs of Hospital Authority’s patients. Data are however available from the authors upon reasonable request and with permission of the Hong Kong Hospital Authority.
