# Correction: A hybrid machine learning framework to improve prediction of all-cause rehospitalization among eldely patients in Hong Kong

**DOI:** 10.1186/s12874-023-01851-6

**Published:** 2023-02-13

**Authors:** Jingjing Guan, Eman Leung, Kin-on Kwok, Frank Youhua Chen

**Affiliations:** 1 Epitelligence, Hong Kong SAR, China; 2 JC School of Public Health and Primary Care, The Chinese University of Hong Kong, Hong Kong SAR, China; 3 Stanley Ho Centre for Emerging Infectious Diseases, The Chinese University of Hong Kong, Hong Kong SAR, China; 4 Hong Kong Institute of Asia-Pacific Studies, The Chinese University of Hong Kong, Hong Kong SAR, China; 5 Department of Management Sciences, City University of Hong Kong, Hong Kong SAR, China

**Correction: BMC Medical Research Methodology 23, 14 (2023)**

**https://doi.org/10.1186/s12874-022-01824-1**

Following publication of the original article [1], Fig. 4 in the original version of this article has been replaced.

The original article has been updated.
