# Supplementary material for: A hybrid machine learning framework to improve prediction of all-cause rehospitalization among elderly patients in Hong Kong
Source: BMC Med Res Methodol. 2023 Jan 13;23:14. doi: 10.1186/s12874-022-01824-1 (PMC9837949; doi:10.1186/s12874-022-01824-1)
Supplement: Supplementary file 1 — Additional file 1. [file 12874_2022_1824_MOESM1_ESM.docx]

**Additional file 1**

The tZIP model is formulated as follows:

$Y_{i}\sim tZIP(p_{i}(\mathbf{Z}_{i},t_{i}),\lambda_{i}(\mathbf{X}_{i},t_{i}),t_{i})$ for the $i$th record with link functions respectively for $p_{i}(\mathbf{Z}_{i},t_{i})$ and $\lambda_{i}(\mathbf{X}_{i},t_{i})$ as

$p_{i}(\mathbf{Z}_{i},t_{i})=\frac{exp(\mathbf{Z}_{i}\boldsymbol{\alpha}+f(t_{i}))}{exp(\mathbf{Z}_{i}\boldsymbol{\alpha}+f(t_{i}))+1}$ and

$\lambda_{i}\left( \mathbf{X}_{i},t_{i} \right)=exp\left( \mathbf{X}_{i}\boldsymbol{\beta}+g\left( t_{i} \right) \right),$

where $\boldsymbol{\alpha}$ and $\boldsymbol{\beta}$ are the regression coefficients to be estimated; $\mathbf{Z}_{i}$ and $\mathbf{X}_{i}$ are two variable vectors of patient attributes for estimating $p_{i}(\mathbf{Z}_{i},t_{i})$ and $\lambda_{i}(\mathbf{X}_{i},t_{i})$; and $p_{i}(\mathbf{Z}_{i},t_{i})$ refers to the likelihood that the patient will be of inactive rehospitalisation status within exposure time $t_{i}$. Meanwhile, if the patient is of active rehospitalisation status${, \lambda}_{i}(\mathbf{X}_{i},t_{i})$ refers to the expected number of future rehospitalisation within exposure time $t_{i}$. Two offset functions $f(t_{i})$ and $g(t_{i})$ were introduced to provide a temporal dimension to $p_{i}$ and $\lambda_{i}$, respectively. The formulation of the tZIP model is a generalisation of the ZIP model, where $p$ (from the logistic component) and $\lambda$ (from the Poisson component) are specified so that they could have nonlinear associations with exposure time. In other words, the tZIP model could degenerate into a ZIP model when $p$ and $\lambda$ do not vary with exposure time.

The offset functions $f(t_{i})$ and $g(t_{i})$ were selected in accordance with their fit with the observed changes in rehospitalisation rate concerning the growing exposure time between discharge and rehospitalisation/end-of-records. Hence, they are data-driven and context-sensitive. Figure S-1 illustrates the rehospitalisation rate of the model-building cohort over 2 years. The horizontal axis in Figure S-1 represents the time between the moment of discharge (time 0) for each record in the cohort and the end of the subsequent 2-year period (i.e., 730 days post-discharge). The vertical axis in Figure S-1 represents the rehospitalisation rate. Each bar in Figure S-1 represents the rehospitalisation rate of the cohort consisting of those who remained in the community until that period since the previous discharge.

As Figure S-1 shows, in contrast to the solid red line representing a constant relationship between rehospitalisation rate and exposure time that most published studies on estimators of rehospitalisation have assumed, the observed rehospitalisation data exhibited a nonlinear increase as the period since discharge grew. Offset functions were introduced to handle such nonlinearity between time and risk. To identify the best fit between the actual data and different clinically feasible formulations of temporal offset functions, we considered a combination of fit statistics, including the Akaike information criterion and Vuong tests (54). Optional offset functions allow $p$ and $\lambda$ to vary in linearity, concavity and convexity against exposure time, including one with no offset, as indicated by the red line in Figure S-1.


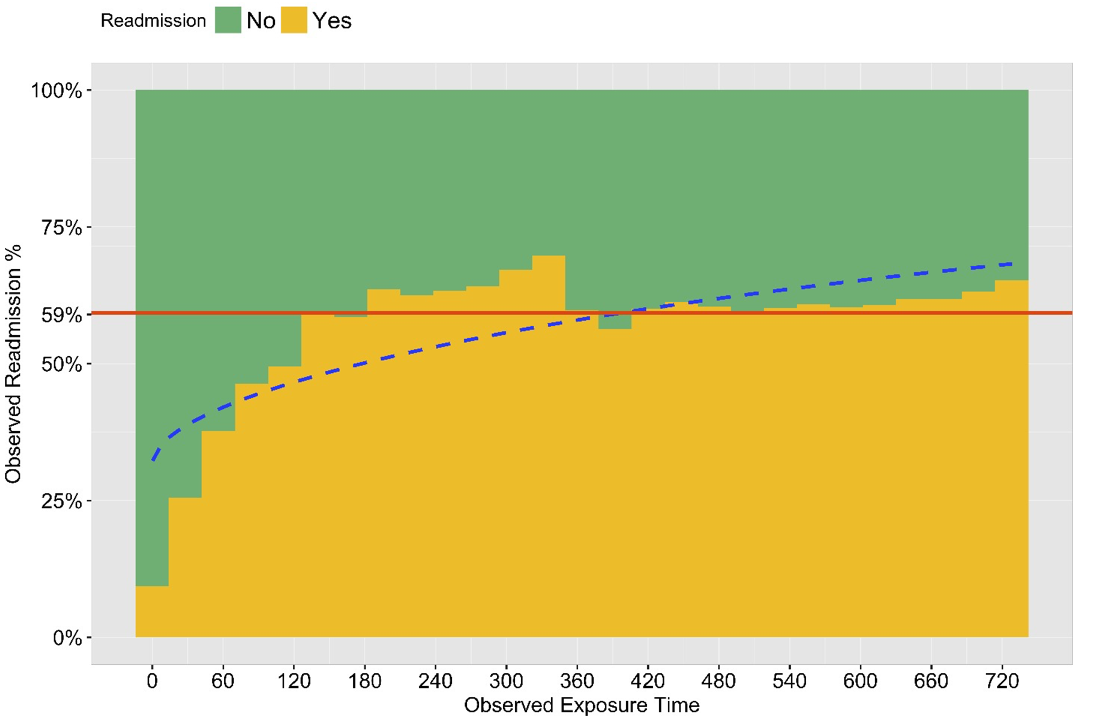


Figure S-1. Distribution of rehospitalisation percentages of the discharged model-building cohort over exposure time in 2 years

A new quantification of readmission risk that joins the two tZIP model-based estimations of rehospitalisation was presented to make full use of the dual-parameter nature of the tZIP regression model in rehospitalisation prediction: the probability of being inactive for rehospitalisation, $\hat{\boldsymbol{p}}\boldsymbol{(}\boldsymbol{t}\boldsymbol{)}$, and the expected rehospitalisation count amongst the high-risk (active) group, $\hat{\boldsymbol{\lambda}}\boldsymbol{(}\boldsymbol{t}\boldsymbol{)}$. Conditional on the regression estimates of the tZIP model, $\hat{\boldsymbol{p}}\boldsymbol{(}\boldsymbol{t}\boldsymbol{)}$and $\hat{\boldsymbol{\lambda}}\boldsymbol{(}\boldsymbol{t}\boldsymbol{)}$, the Joint Estimator (JE), which was derived from each index hospitalisation record given its respective exposure time, could be defined as follows:

$$\text{JE}=\left\{ \begin{matrix} \text{Expected readmission counts at data extraction date,} & \text{ exposure time within }\text{r}\text{ days} \\ \left( expected readmission counts when the exposure time is r days \right)\text{ -1}, & \text{ exposure time beyond }\text{r}\text{ days.} \end{matrix} \right.$$

Given the offsets introduced above, $\hat{\boldsymbol{p}}(\boldsymbol{t})$and $\hat{\boldsymbol{\lambda}}(\boldsymbol{t})$ were changed continuously as a function of the exposure time whilst $\hat{\boldsymbol{p}}(\boldsymbol{t})$and $\hat{\boldsymbol{\lambda}}(\boldsymbol{t})$ were integrated such that JE = $\hat{\boldsymbol{p}}\left( \boldsymbol{t} \right)\cdot\boldsymbol{0}+\left( \boldsymbol{1}-\hat{\boldsymbol{p}}\left( \boldsymbol{t} \right) \right)\hat{\boldsymbol{\lambda}}\left( \boldsymbol{t} \right)$. JE is an estimation of the *expected number of future rehospitalisation* at any given moment during the exposure time. As the exposure time $(\boldsymbol{t})$ approaches *r*, JE is reset, making it a discontinuous function of time.

Figure S-2 below illustrates the process in which the hybrid decision tree algorithm applies URPSS to supervise learning from an EHR database:

1. **Input:** an acute inpatient cohort’s dataset
2. **Initial Step:**

- Features with no variability were removed.
- Across features, a global null hypothesis of independence between all features and rehospitalisation was tested.
- If rejected, the partition returned the feature with the strongest association with the rehospitalisation outcome. If it was a continuous variable, the partition returned a cut-off value for the feature; otherwise, it returned two partitioned subsets.
- The original dataset was split into two sub-datasets by the identified feature and its cut-off.

**14. Iteration:** The initial step was repeated in the two sub-datasets.

1. **Stopping Rule:** When the global null hypothesis of independence could not be rejected or when no more variability of effects was present amongst features within a sub-dataset, the iteration would be stopped.
2. **A hybrid decision tree of URPSS as the final output:** Optionality of post-acute care led to missing values in features, where URPSS assigned 0 weight to observations of missing values and allowed surrogate splits to establish a binary partition of all observations in the sub-dataset.
3. **Interpretation of results:** The sequence where features were selected through URPSS reflected the ‘importance’ (61) of the feature with respect to the supervisory variable, which was 28-day rehospitalisation in this case. In the decision tree diagram reported in Figure S-2, the higher the features along the vertical axis, the greater the ‘importance’ of the feature regarding 28-day readmission.


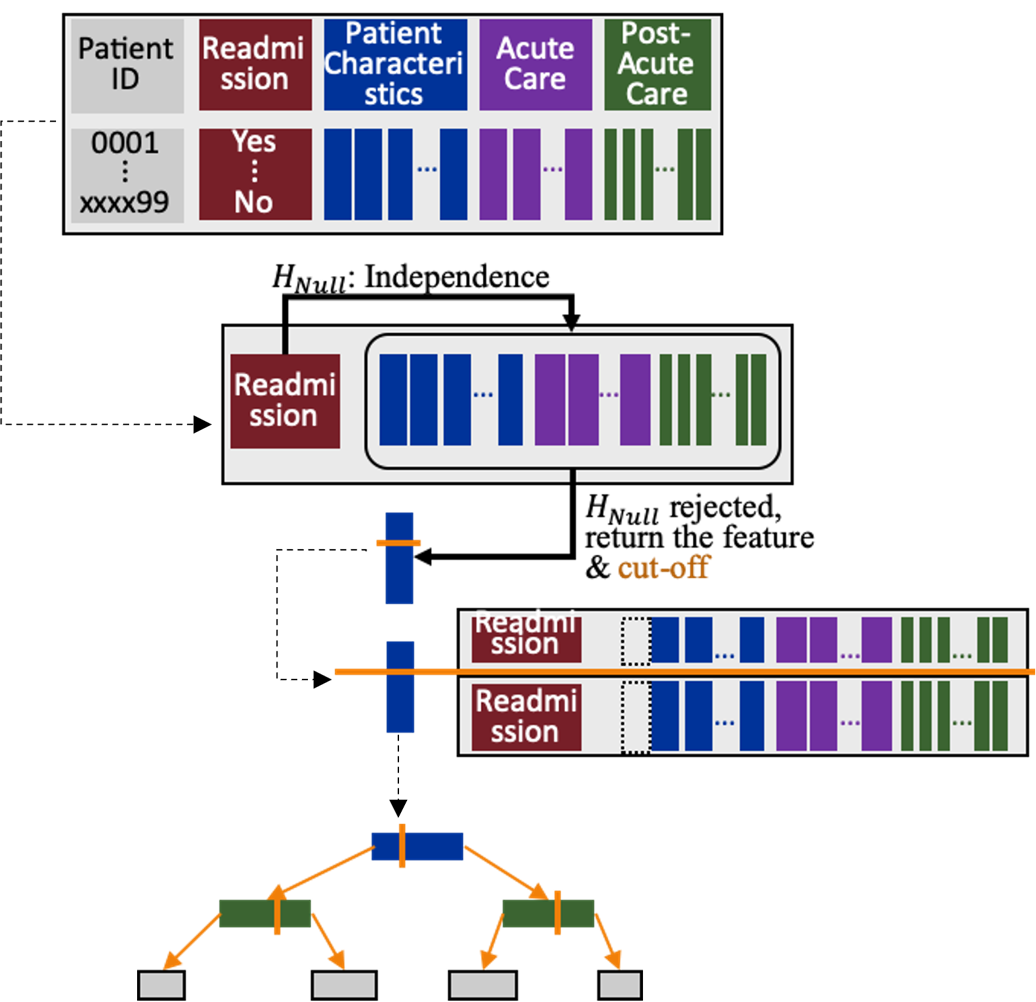


Figure S-2. Schematic of ranking features’ marginal importance with the hybrid machine learning model
